# Shape Memory and Mechanical Properties of Cold Rolled and Annealed Fe-17Mn-5Si-5Cr-4Ni-1Ti-0.3C Alloy

**DOI:** 10.3390/ma14020255

**Published:** 2021-01-06

**Authors:** Dohyung Kim, Kinam Hong, Jeesoo Sim, Junghoon Lee, Wookjin Lee

**Affiliations:** 1 Dongnam Division, Korea Institute of Industrial Technology, Yangsan 50623, Korea; dhyungkim@kitech.re.kr (D.K.); simexp95@kitech.re.kr (J.S.); 2 Department of Material Science and Engineering, Pusan National University, Busan 46241, Korea; 3 Department of Civil Engineering, Chungbuk National University, Chungju 28644, Korea; hong@chungbuk.ac.kr; 4 Department of Metallurgical Engineering, Pukyong National University, Busan 48513, Korea

**Keywords:** shape memory alloy, Fe-Mn-Si, microstructure, mechanical properties

## Abstract

In the present study, the shape, memory, and mechanical properties of cold-rolled and annealed Fe-17Mn-5Si-5Cr-4Ni-1Ti-0.3C (wt.%) alloy were investigated. The cold-rolled alloy was annealing heat-treated at different temperatures in the range of 500–900 °C for 30 min. The shape recovery behavior of the alloy was investigated using strip bending test followed by recovery heating. The microstructural evolution and the stress-strain response of the alloy heat-treated at different temperatures revealed that the recovery took place at a heat-treatment temperature higher than 600 °C. Recrystallization occurred when the heat-treatment temperature was higher than 800 °C. Meaningful shape recovery was observed only when the alloy was annealed at temperatures higher than 600 °C. The highest recovery strain of up to 2.56% was achieved with a pre-strain of 5.26% and recovery heating temperature of 400 °C, when the alloy was heat-treated at 700 °C. Conversely, the yield strength reduced significantly with increasing annealing heat-treatment temperature. The experimental observations presented in this paper provide a guideline for post-annealing heat-treatment when a good compromise between mechanical property and shape recovery performance is required.

## 1. Introduction

Fe-Mn-Si-based shape memory alloys (Fe-SMAs) are steel-based alloys that exhibit the unique and characteristic behavior of shape memory effect. The shape memory effect of this alloy system was first discovered by Sato et al. in 1982 [1]. Since then, numerous studies have been conducted to use the alloys in structural applications due to their high elastic stiffness; strength; and, most importantly, low raw material cost. One possible application of the alloys could be the use of their high recovery-induced stress. By restraining the shape recovery, a so-called recovery stress can be generated in the material, resulting in pre-stressing or clamping forces [2]. Such behavior can be used in pipe joints, rail couplings, or pre-stressing reinforcement of civil structures [3,4,5,6,7,8,9,10]. The recovery stress ranging from 130 to 580 MPa has been reported with various heating temperatures between 130 and 580 °C on different Fe-SMAs [11]. It has been shown that Fe-SMA has good fatigue [12] and corrosion resistance [13], making it even more attractive in structural applications.

The behavior of shape memory in the Fe-SMA is associated with the stress-induced γ-austenite (face-centered cubic) to ε-martensite (hexagonal close-packed) phase transformation and its reverse during heating [7]. In recent years, various Fe-SMAs have been under intense investigation. Most of the alloys investigated were recently characterized by microstructures with fine ceramic precipitates, e.g., NbC [14,15], VN [16], or VC [6,17]. The fine precipitates are known to make the γ to ε phase transformation easier, thereby significantly improving the alloy’s shape recovery performance [10]. Post-aging heat-treatments have been carried out in these alloy systems to disperse the fine precipitates in the Fe-SMA microstructures. On the other hand, a new type of Fe-SMA containing TiC precipitate has recently been reported by Hong et al. [18]. In this alloy, fine TiC precipitates were well dispersed in the microstructure after a hot-rolling process without any post-aging heat-treatment. The alloy exhibited a good shape memory effect with a recovery strain of near 1% and a recovery stress of 518 MPa, while the alloy was pre-strained by 4% and heated to 200 °C. It was also found that the alloy has good mechanical properties with a tensile yield strength of near 600 MPa and a fatigue limit of 473 MPa, without post-aging heat-treatment [19].

The present study is a continuation of the studies conducted by Hong et al. [18,19]. As noted by Arabi-Hashemi et al. [10], the yield strength is the main limiting factor to achieving significant recovery stress. In previous studies, the hot-rolled Fe-SMA containing TiC demonstrated a tensile yield strength of approximately 600 MPa. In this study, cold-rolling and annealing heat-treatment were additionally performed to further increase the Fe-SMA yield strength. The same hot-rolled Fe-SMA described previously [18,19] was used as the starting material. The microstructure, shape memory behavior, and mechanical properties of the cold-rolled and annealed Fe-SMA containing TiC were investigated.

## 2. Materials and Methods

The Fe-SMA used in this study consisted of the chemical composition listed in Table 1. The Fe-SMA samples were fabricated by first producing a 50 Kg ingot using a vacuum-induction melting device (Hankook Vacuum Metallurgy Co., Ltd., Ansan, Republic of Korea). The ingot underwent homogenization and heat-treatment at 1250 °C for 6 h. After the homogenization treatment, the alloy ingot was forged and stepwisely hot-rolled at 1000 °C until the alloy’s thickness reached 5 mm. Finally, the hot-rolled alloy was cold-rolled at room temperature (RT, ~25 °C) in a single pass via a 10% reduction in thickness, i.e., to a thickness of 4.5 mm. The thickness reduction of 10% was selected based on preliminary tests where cold-rolling of this alloy caused a thickness reduction of more than 10%, resulting in cracks.

Samples for tensile and bending tests were cut from cold-rolled Fe-SMA using wire electric discharge machining heat-treatment. Machined samples were heat-treated at 500, 600, 700, 800, and 900 °C for 30 min and air cooled to characterize the effect of heat-treatment followed by cold-rolling. Samples were wrapped with steel foil to prevent oxidation during heat-treatment. The heat-treated samples were named 500-HT, 600-HT, 700-HT, 800-HT, and 900-HT, respectively, where 500, 600, 700, 800, and 900 represented the heat-treatment temperatures.

Microstructural characterization of Fe-SMA samples was performed using an optical microscope (OM, Eclipse E200, Nikon, Tokyo, Japan) and scanning electron microscope (SEM, JSM-7200F, JEOL Inc., Tokyo, Japan), equipped with energy dispersive spectrometry (EDS, X-Max^N^, Oxford Instrument, Abingdon, UK). Electron backscatter diffraction (EBSD, Nordlysnano, Oxford Instrument) was used to investigate phase characterization and texture evolution. The samples for the microstructural analyses were mechanically ground using SiC papers and subsequently polished with alumina and 0.04 μm silica colloidal suspensions. The samples were color-etched by 0.5% NH_4_HF_2_ and 1.2% K_2_S_2_O_5_ (*w*/*w*) solution.

Microhardness was measured by the Vickers hardness tester (HM-220A, Mitutoyo Corp., Kawasaki, Japan) under a load of 0.1 kgf. The hardness measurements were performed seven times for each specimen. The alloy’s stress-strain curves were characterized using an uniaxial tensile testing machine (UNITECH-T, R&B Inc., Daejeon, Republic of Korea) with a KS-B801 standard [20]. The total length, gauge length, and thickness of the tensile specimen were 27.2 mm, 13.2 mm, and 2 mm, respectively. For each specimen, at least two tests were performed, which resulted in almost identical results.

**Figure 1:**
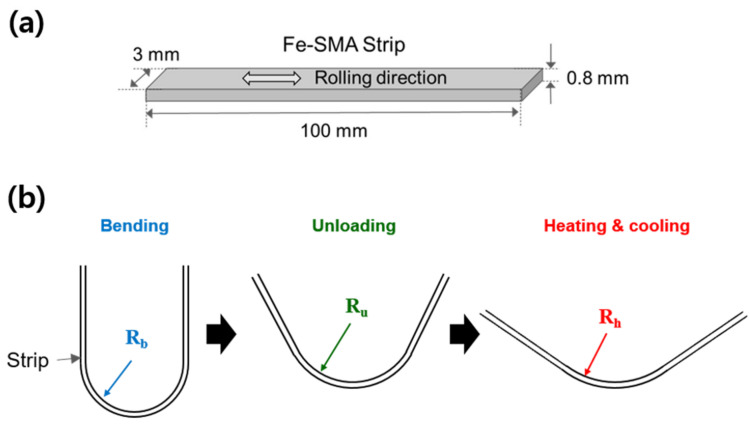
Schematics of bending tests: (**a**) geometry of the bending strip, and (**b**) strip bending and recovery test.

The shape recovery performance of the alloy was investigated by the strip bending and recovery test. Figure 1a shows schematically the bending strip used in the study. Strip-shaped specimens with a length of 100 mm, a width of 3 mm, and a thickness of 0.8 mm were cut along the cold-rolling direction from the middle of the cold-rolled plate. The bending and recovery test procedure is shown schematically in Figure 1b. For the bending test, the specimen was bent around a mold with a radius of 7.2 mm at RT, applying approximately 5.26% bending pre-strain to the specimen, as described previously [21]. The bending radius of the strip at its neutral plane was 7.6 mm, which is evaluated by the formula $R_{m}+0.5\cdot t$ where *R_m_* is the radius of the bending mold. The pre-strain, *ε_pre_*, was taken as the maximum tensile strain at the bottom surface of the strip thickness, and is determined as follows:

(1) $$\varepsilon_{pre}=\frac{t}{2\cdot R_{b}}$$

where *R_b_* is the bending radius and *t* is the strip thickness [21]. The pseudoelastic strain, *ε_pse_*, was determined by measuring the final bend radius, *R_u_*, after unloading as expressed in Equation (2) below:

(2) $$\varepsilon_{pse}=\varepsilon_{pre}-\frac{t}{2\cdot R_{u}},$$

The bent strip was then recovery-annealed at different temperatures in a range of 150–400 °C for 30 min for shape recovery. The recovery strain, *ε_rec_*, was calculated by the following equation:

(3) $$\varepsilon_{pse}=\varepsilon_{pre}-\varepsilon_{pse}-\frac{t}{2\cdot R_{h}},$$

where *R_h_* is the bending radius of the strip after the heating and cooling cycle, as shown schematically in Figure 1b. The evaluations of the radii, *R_u_* and *R_h_*, of the specimens were carried out by measuring the angle between the two straight ends and using the following equation:

(4) $$\pi \cdot R_{b}=\left(\pi -\theta_{u}\right)\cdot R_{u}=\left(\pi -\theta_{h}\right)\cdot R_{h}$$

where *θ_u_* and *θ_h_* are the angles between the two ends of the bent strips, after unloading and after recovery heating, respectively. The angles were measured by a digital protractor with a resolution of 0.01°. Before the main tests were performed, the reproducibility of the bending and recovery tests was accessed by repeating the test three times with the as-rolled strips. The standard deviation of the *R_u_* and *R_h_* between the three individual tests for the as-rolled strips was less than 0.1%.

## 3. Results and Discussions

### 3.1. Microstructures

**Figure 2:**
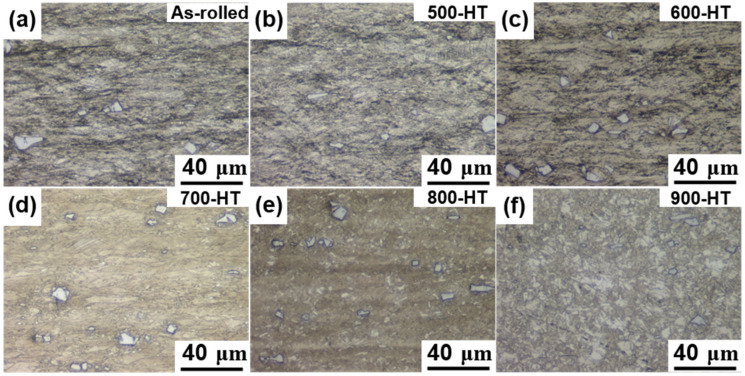
Optical microscope (OM) images (×500) of Fe-Mn-Si-based shape memory alloys (Fe-SMA) in different heat-treatment conditions. Rolling direction is horizontal to the images; (**a**) as-rolled, (**b**) 500 °C heat-treated, (**c**) 600 °C heat-treated, (**d**) 700 °C heat-treated, (**e**) 800 °C heat-treated, and (**f**) 900 °C heat-treated.

**Figure 3:**
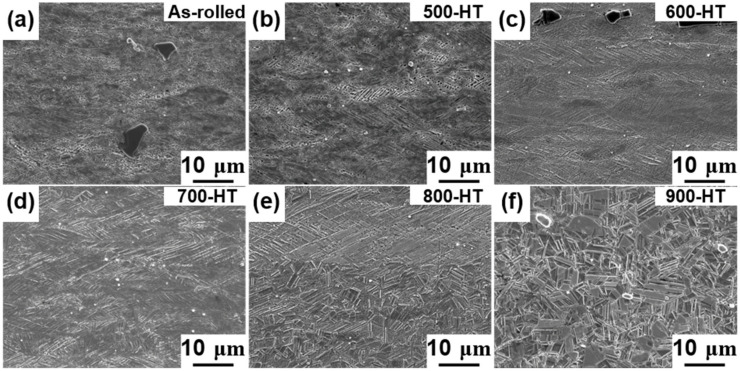
Scanning electron microscope (SEM) images (×2000) of Fe-SMA in different heat-treatment conditions. Rolling direction is horizontal to the images; (**a**) as-rolled, (**b**) 500 °C heat-treated, (**c**) 600 °C heat-treated, (**d**) 700 °C heat-treated, (**e**) 800 °C heat-treated, and (**f**) 900 °C heat-treated.

Figure 2 and Figure 3 illustrate the OM and SEM images of the Fe-SMAs in the as-rolled and annealing heat-treated conditions. In the OM images, the γ-phases appear in brown, the ε-phases appear in white, and the α’-(body-centered tetragonal) phases appear in dark blue, [7]. The ε-phases in the SEM images appear as thin needle-like shapes for deformed grain and plate-like shapes for recrystallized grains. The as-rolled microstructure consisted of highly deformed grains. Shear bands were clearly seen in the microstructure, which were tilted by approximately 45 degrees to the rolling direction (RD). It was obvious that the shear bands gradually became wider with an increase in annealing temperature ranging from 500 to 800 °C, as shown in Figure 2b–e and Figure 3b–e. The microstructure was mainly composed of highly deformed γ-grains in the as-rolled state and remained unchanged when the annealing temperature was less than 600°C, as shown in Figure 2a–c. When the annealing temperature was above 700 °C, a small amount of ε-phase was also observed in the microstructure. The amount of ε-phase increased gradually with increasing annealing temperature, as shown in Figure 2d–f.

After annealed at 800 °C, the Fe-SMA was partially recrystallized, with the average grain size in the recrystallization regions being approximately 5 μm, as shown in Figure 2e. When the annealing temperature was 900 °C, the Fe-SMA was fully recrystallized, as can be seen in Figure 2f. The recrystallization in the annealed Fe-SMA occurred in the form of new equiaxed grains. It can also be observed that the recrystallized grains contain a large number of annealing twins, which is typical for the Fe-SMA [10].

**Figure 4:**
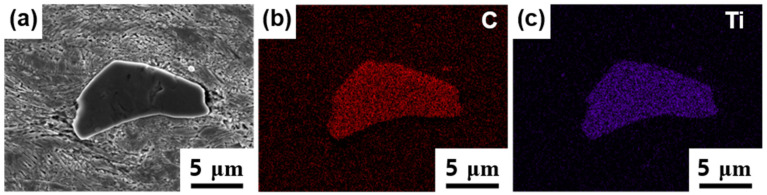
Energy dispersive spectrometry (EDS) chemical composition maps near a precipitate in as-rolled Fe-SMA sample. (**a**) SEM image of mapped area, (**b**) map of Carbon (C), and (**c**) Titanium (Ti).

As shown in Figure 2 and Figure 3, the Fe-SMA microstructure consisted of precipitates regardless of annealing treatments. Figure 4 depicts the EDS maps obtained near the precipitate of the as-rolled sample, showing the dominant elemental distributions of the precipitate. The elemental distribution map in Figure 4 confirmed the presence of TiC precipitates with a rich presence of titanium (Ti) and carbon (C) compared to the surrounding matrix.

**Figure 5:**
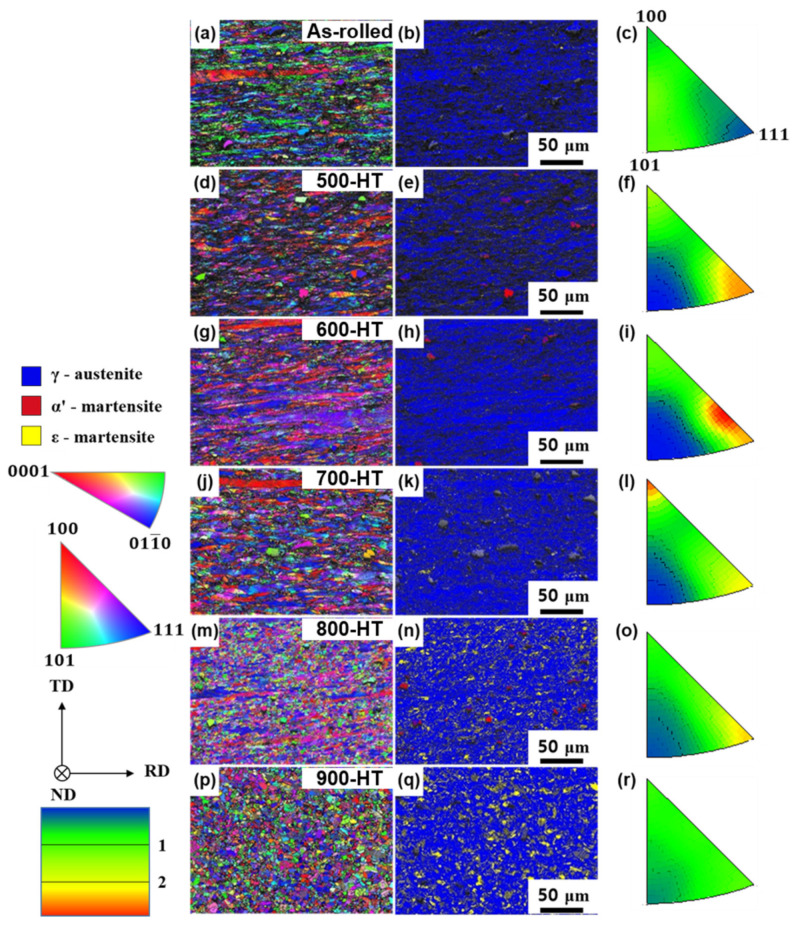
(**a**,**d**,**g**,**j**,**m**,**p**) Electron backscatter diffraction (EBSD) rolling direction (RD) inverse pole figure maps, (**b**,**e**,**h**,**k**,**n**,**q**) phase maps, and (**c**,**f**,**i**,**o**,**r**) RD pole figures; (**a–c**) as-rolled, (**d–f)** 500 °C heat-treated, (**g–i**) 600 °C heat-treated, (**j–l**) 700 °C heat-treated, (**m–o**) 800 °C heat-treated, and (**p–r**) 900 °C heat-treated.

EBSD inverse pole figure maps, phase maps, and pole figures are shown in Figure 5 for the as-rolled and annealing heat-treated samples. In the as-rolled state, the {101}//RD texture component was developed; however, the texture was weak, with a maximum intensity of 1.2. The samples annealed at 500, 600, and 700 °C showed strong {111}//RD and {100}//RD textures. As previously mentioned, these samples retained the original deformed γ-grains with a slight grain reorientation due to the extension of the shear bands, without any pronounced recrystallization. Hence, the strong {111}//RD and {100}//RD textures in these samples were presumably generated due to the cold-rolling process. Textures of the sample annealed at 800 °C were weakened through recrystallization compared with the sample annealed at 700 °C, as can be seen in Figure 5o. The intensities of the textures decreased further in the sample annealed at 900 °C, confirming the elimination of texture by recrystallization.

As expected from the OM results, small ε-grains formed in the microstructure when the annealing temperature was higher than 700 °C, as shown in Figure 5. Table 2 lists the EBSD phase fraction measurement results for the as-rolled and heat-treated Fe-SMAs. The amount of the ε-phase increased with the annealing temperature, indicating the formation of thermally induced ε-martensite during recrystallization. The EBSD maps for the as-rolled and 500-HT samples consisted of large fractions of non-indexed area due to the severe plastic deformation occurring during the cold-rolling process. The non-indexed area in the EBSD phase map decreased with increasing annealing temperature as the annealing of the lattice damage caused by the cold-rolling took place, as shown in Table 2.

### 3.2. Shape Recovery Behavior

**Figure 6:**
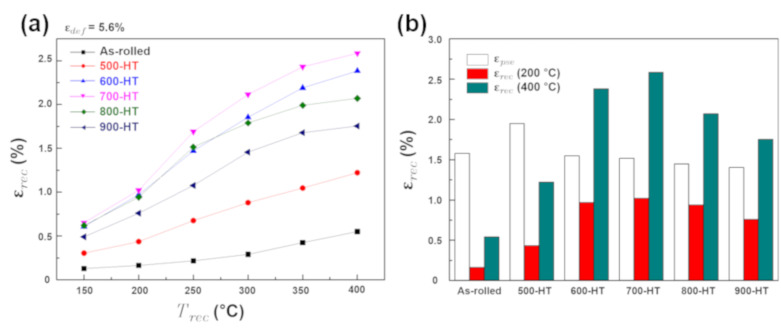
(**a**) Evolutions of shape recovery strains with heating temperatures for different heat-treatment conditions; and (**b**) pseudoelasticity, and shape recovery strains, at 200 °C heating and 400 °C heating.

Figure 6a shows the *ε_rec_* as a function of the recovery heating temperature (*T_rec_*) for as-rolled and annealing heat-treated samples, for the samples bent by *R_b_* = 7.6 mm (*ε_pre_* = ~5.26%). The as-rolled sample showed only a small shape recovery, i.e., less than 0.5% while heating to 400 °C. This data indicates that microstructural defects such as dislocations and shear bands produced during severe plastic deformation by cold-rolling suppressed the γ to ε phase transformation. A similar observation was also made in the case of severely plastic-deformed Ni-Ti-based shape memory alloy [22]. When samples were annealing heat-treated, a pronounced shape recovery took place, as shown in Figure 6a. The shape recovery started to occur at a temperature lower than 150 °C. This means that the austenite starting temperature (As) is far lower than 150 °C. As demonstrated in a previous study [18], the onset of this alloy’s shape recovery took place immediately upon heating from RT, indicating that the As of the alloy is lower than RT. The recovery strain increased gradually by increasing the recovery heating temperature up to 400 °C in all the samples tested. These results suggest that the austenite finishing temperature (Af) of Fe-SMA, i.e., the temperature where ε to γ phase transformation finishes upon heating with no stress, is above 400 °C. An Af temperature above 400 °C is significantly higher than previously studied alloys. As a comparison, the Af temperatures for the Fe-SMAs containing NbC [15] and VC [6] are about 300 and 175 °C, respectively. The high Af temperature of the Fe-SMA containing TiC can provide wide temperature hysteresis, which is desirable for applications aimed at permanent recovery stress [5].

When the annealing temperature was increased, the *ε_rec_* increased sharply initially and then decreased. This reaction can be more clearly seen in Figure 6b, where the *ε_rec_* for *T_rec_* = 200 and 400 °C is compared between the as-rolled and annealing heat-treated samples. For *T_rec_* = 200 °C, the recovery strain was less than 0.3% in the as-rolled state. The *ε_rec_* was increased to 0.43 and 0.97% when the samples were annealed at 500 and 600 °C, respectively. The sample annealed at 700 °C revealed the highest *ε_rec_* of 1.02%. The recovery strain of the 600-HT and 700-HT samples for *T_rec_* = 200 °C were comparable to the recovery strain of the hot-rolled sample [18]. However, a one-to-one comparison was not possible due to the different test configurations, i.e., the present study used bending tests with a pre-strain of 5.26% while the previous study used tensile tests with a pre-strain of 4%. When the annealing temperature was higher than 800 °C, the *ε_rec_* moderately decreased with increasing annealing temperature. The evolution of the *ε_rec_* for *T_rec_* = 400 °C follows the same trend as the *ε_rec_* for *T_rec_* = 200 °C but with a much higher recovery of up to 2.5%. The 700-HT sample again showed the highest recovery strain of 2.56%.

The decreasing trend of the recovery strain with increasing annealing temperature over 700 °C can be interpreted in terms of increasing the thermally induced ε-martensite phases in the microstructure, as they cannot produce the recovery strain nor the reversible γ to ε phase transformation. The texture effect does not seem prominent in determining the recovery strain since {100} and {111}—the strong texture components in the 700-HT sample that were weakened while further increasing the annealing temperature—are less favorable than {101} for the γ to ε phase transformation [23].

In Figure 6b, the *ε_pse_* is also depicted for the as-rolled and annealing heat-treated samples. The *ε_pse_* was around 1.5%, except for the 500-HT sample where a high pseudoelastic strain of nearly 2% was obtained. If the material follows an elastic-perfect plastic behavior, the elastic spring-back of the bent strip can be estimated by the following equation [24]:

(5) $$\frac{R_{b}}{R_{u}}=1-3\cdot \left(\frac{\sigma_{y}\cdot R_{b}}{E\cdot t}\right)+4\cdot {\left(\frac{\sigma_{y}\cdot R_{b}}{E\cdot t}\right)}^{2},$$

where *σ_y_* is the yield strength, *t* is the thickness of the strip, and *E* is the elastic modulus. Assuming *R_b_* = 7.2 mm, *E* = 200 GPa, and *t* = 0.8 mm, the calculated *R_u_* values by the Equation (4) are 7.8 and 8.0 mm for *σ_y_* = 600 and 800 MPa, respectively. Considering Equation (2), the pure elastic spring-back of the strips in a given test condition is around 0.5%, which is 3–4 times smaller than the measured *ε_pse_* values of 1.5–2%. Thus, the results indicate significant pseudoelastic behavior of the Fe-SMA containing TiC.

### 3.3. Mechanical Properties

**Figure 7:**
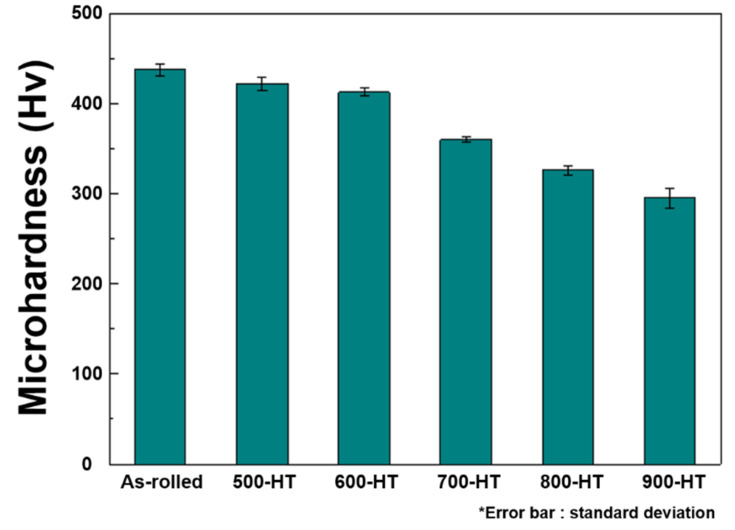
Evolution of microhardness with increasing heat-treatment temperature.

**Figure 8:**
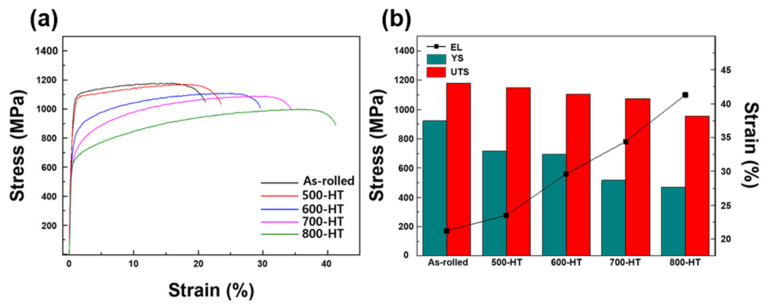
(**a**) Stress-strain curves and (**b**) yield strength (YS), ultimate tensile strength (UTS), and maximum elongation (EL) of Fe-SMA with different heat-treatment conditions.

Figure 7 shows the evolution of microhardness with increasing annealing heat- treatment temperature. The results of hardness measurements showed a gradually decreasing trend of the hardness with the annealing temperature. The tensile properties of the Fe-SMAs for the as-rolled and annealing heat-treated conditions are depicted in Figure 8. In the as-rolled condition, the alloy showed a typical stress-strain response of strain-hardened steel, i.e., clear yielding followed by plastic deformation. The yield strength of the as-rolled Fe-SMA was over 900 MPa, which was much higher than the strength of approximately 600 MPa of the same hot-rolled alloy [18]. This data indicates the strong work-hardening capability of the Fe-SMA containing TiC. Increasing the annealing temperature resulted in decreased yield and tensile strengths while the maximum elongation was increased, as shown in Figure 8b. This behavior can be easily understood by considering the annealing and recrystallization. During the annealing heat-treatment, microstructural defects such as dislocations and shear bands are released, leading to the easier γ to ε phase transformation and γ slip deformation. When the annealing heat-treatment temperature is higher than 800 °C, high density dislocation develops into recrystallization nucleation, as shown in Figure 3. These two mechanisms reduce interactions between dislocations and hence lower the strength of the alloy. Meanwhile, the 500-HT and 600-HT samples maintained comparably high-yield strengths of around 700 MPa, which are still higher by approximately 100 MPa compared to the hot-rolled Fe-SMA [18]. For these annealing conditions, the texture effect works by increasing the yield strength since the {101} texture component decreases where the strength is the weakest, as reported by Arabi-Hashemi et al. [10]. As shown in Figure 5, the {101} texture intensity along the RD increased with annealing temperature when the annealing temperature was higher than 700 °C, following the recrystallization. The low yield strengths in the samples annealed at over 700 °C are probably associated with the texture effect as well as with the replacement of severely work hardened structures by undistorted equiaxed grains by the recrystallization.

Considering the application of this alloy as a prestressing reinforcement of concrete structures, cold-rolling and annealing at 500 or 600 °C seem to be effective in obtaining significant recovery stress. As shown in Figure 6b, the recovery strain of the 500-HT and 600-HT samples were approximately 0.5 and 1%, respectively, for *T_rec_* = 200 °C. These recovery strains are comparably small but significant enough to produce large recovery stress [10]. As the yield strength is the main limiting factor in obtaining significant recovery stress, high yield strengths of around 700 MPa in the above annealing conditions would be favorable for such applications. In these annealing conditions, a good combination of strength (near 700 MPa) and ductility (higher than 20%) were obtained. On the other hand, the largest shape recovery could be obtained by annealing the alloy at 700 °C. In this condition, the Fe-SMA exhibited a large recovery strain of up to 2.56% for *T_rec_* = 400 °C, comparably low yield strength of approximately 500 MPa, and excellent ductility (over 30%). This annealing condition would be favored for applications where a large shape recovery is required, e.g., for joint components or pipe clamping.

## 4. Summary

In the present study, the shape memory and mechanical properties of cold-rolled and annealed Fe-17Mn-5Si-5Cr-4Ni-1Ti-0.3C (wt.%) alloy were investigated. Based on the experimental findings, the following conclusions were derived:

1. The Fe-SMA containing TiC showed a strong work hardening capability. The Fe-SMA cold-rolled to 10% reduction of the thickness exhibited a yield strength over 900 MPa, which is more than 1.5 times larger than the strength of the same hot-rolled alloy.
2. The alloy in the as-rolled state exhibited very small shape recovery, i.e., less than 0.5% while heating to 400 °C. This result indicates that microstructural defects such as dislocations and shear bands produced during the severe plastic deformation by cold-rolling suppressed the γ to ε phase transformation. When the heat-treated alloy was annealing heat-treated, pronounced shape recovery took place. Increasing the annealing temperature resulted in a sharp increase in the shape recovery and then a decrease due to recrystallization.
3. When the alloy was annealing heat-treated at a temperature range of 500–700 °C, there were strong {111}//RD and {100}//RD crystallographic textures due to the severe cold-rolling process. The intensities of the textures decreased when the alloy was annealed at above 800 °C due to the recrystallization. However, the texture effect was not prominent in determining the recovery strain.
4. The hardness and the yield strength of the alloy gradually decreased with increasing annealing temperature. In reviewing the application of this alloy as a prestressing reinforcement of concrete structures, annealing at 500 or 600 °C seems to be effective in obtaining a large recovery stress. In these annealing conditions, a good combination of yield strength near 700 MPa and ductility higher than 20% are obtained.
5. The largest shape recovery was obtained by annealing the alloy at 700 °C. In this condition, the Fe-SMA exhibited a large recovery strain of up to 2.5%, comparably low yield strength of approximately 500 MPa, and excellent ductility over 30%. This annealing condition would be favored for the applications where a large shape recovery is required, e.g., for joint components or pipe clamping.

## Figures and Tables

**Table 1:** Chemical composition of Fe-Mn-Si-based shape memory alloys (Fe-SMA).

Elements	Fe	Mn	Si	Cr	Ni	Ti	C
Contents (wt.%)	balance	17	5	5	4	1	0.3

**Table 2:** EBSD phase fraction results of Fe-SMAs.

EBSD Phase Fraction (%).	As-Rolled	500-HT	600-HT	700-HT	800-HT	900-HT
γ-austenite	37.52	32.00	53.71	58.54	63.52	65.51
ε-martensite	0.16	0.72	0.29	0.78	8.81	10.86
α′-martensite	0.86	0.7	0.54	1.18	0.71	0.57
Non-indexed	61.45	66.55	45.45	39.50	26.96	23.05

## Data Availability

The data presented in this study are available on request from the corresponding author, upon reasonable request.
